# Evaluation of vestibular evoked myogenic potentials (VEMP) and electrocochleography for the diagnosis of Ménière's disease^☆^

**DOI:** 10.1016/j.bjorl.2016.04.021

**Published:** 2016-06-02

**Authors:** Pauliana Lamounier, Thiago Silva Almeida de Souza, Debora Aparecida Gobbo, Fayez Bahmad Jr.

**Affiliations:** a Universidade de Brasília (UNB), Ciências da Saúde, Brasília, DF, Brazil; b Centro de Reabilitação e Readaptação Dr. Henrique Santillo (CRER-GO), Goiânia, GO, Brazil; c Pontifícia Universidade Católica de Goiás (PUC-GO), Goiânia, GO, Brazil; d Instituto Brasiliense de Otorrinolaringologia, Brasília, DF, Brazil

**Keywords:** Ménière's disease, Electrocochleography, Vestibular evoked myogenic potential, Doença de Ménière, Eletrococleografia, Potencial evocado miogênico vestibular

## Abstract

**Introduction:**

Ménière's disease (MD) is an inner ear disorder characterized by episodic vertigo, tinnitus, ear fullness, and fluctuating hearing. Its diagnosis can be especially difficult in cases where vestibular symptoms are present in isolation (vestibular MD). The definitive diagnosis is made histologically and can only be performed *post-mortem*, after analysis of the temporal bone. Endolymphatic hydrops is a histopathological finding of the disease and occurs more often in the cochlea and saccule, followed by the utricle and semicircular canals. Vestibular evoked myogenic potentials (VEMP) emerged as the method of assessment of vestibular function in 1994. Until then, there was no unique way of assessing saccular function and the inferior vestibular nerve. Given that the saccule is responsible for most cases of severe hydrops, VEMP appears as a new tool to assist in the diagnosis of MD.

**Objective:**

To evaluate the sensitivity and specificity of VEMP and electrocochleography (EcochG) in the diagnosis of definite MD compared with clinical diagnosis.

**Methods:**

The study includes 12 patients (24 ears) diagnosed with definite MD defined according to the clinical criteria proposed by the American Academy of Otolaryngology – Head and Neck Surgery (AAO-HNS) in 1995, as well as 12 healthy volunteers allocated to the control group (24 ears). A clinical diagnosis by the AAO-HNS criteria was considered as the gold standard. All patients underwent an otoneurological examination, including pure tone and speech audiometry, VEMP, and extratympanic EcochG. The sensitivity and specificity to detect the presence or absence of disease were calculated, as well as their 95% confidence intervals. The reliability of VEMP and EcochG in both ears was assessed using the kappa index.

**Results:**

In both tests and in both ears, the ability to diagnose healthy cases was high, with specificity ranging from 84.6% to 100%. Moreover, the ability of the tests to diagnose the disease varied from low to moderate sensitivity, with values ranging from 37.5% to 63.6%. The agreement of both tests in the right ear, measured by the kappa coefficient, was equal to 0.54 (95% CI: 0.20–0.89), indicating a moderate agreement. In the left ear, that agreement was equal to 0.07 (95% CI: −0.33 to 0.46), indicating a weak correlation between the tests. The sensitivity of the VEMP for the right ear was 63.6% and for the left ear, 62.5%. The sensitivity of EcochG for the right ear was 63.6% and 37.5% for the left ear.

**Conclusion:**

The specificity of both tests was high, and the sensitivity of VEMP was higher than that of EcochG.

## Resumo

**Introdução:**

A Doença de Ménière (DM) é uma doença da orelha interna caracterizada por vertigem episódica, zumbido, plenitude aural, e audição flutuante. Seu diagnóstico pode ser especialmente difícil nos casos em que os sintomas vestibulares estão presentes isoladamente (DM vestibular). O diagnóstico de certeza é histológico e somente pode ser realizado no *post mortem*, após análise do osso temporal. A hidropisia endolinfática é um achado histopatológico da doença e ocorre mais frequentemente na cóclea e sáculo, seguidos pelo utrículo e canais semicirculares. Os potenciais evocados miogênicos vestibulares (VEMP) surgiram como método de avaliação da função vestibular desde 1994. Até então não havia uma maneira exclusiva de avaliação da função sacular e do nervo vestibular inferior e sendo o sáculo responsável por grande parte dos casos de hidropisia severa, o VEMP aparece como uma nova ferramenta para auxiliar no diagnóstico da DM.

**Objetivo:**

Avaliar a sensibilidade e especificidade do VEMP e da Eletrococleografia (ECochG) no diagnóstico da DM em comparação com o diagnóstico clínico.

**Método:**

: Foram selecionados 12 pacientes (24 orelhas) com diagnóstico de DM definida de acordo com os critérios clínicos propostos pela *American Academy of Otolaryngology-Head and Neck Surgery* 1995 (AAO-HNS) e 12 voluntários saudáveis alocados no grupo controle (24 orelhas). Considerou-se o diagnóstico clínico pela AAO-HNS como padrão ouro. Todos os pacientes foram submetidos a exame otoneurológico, incluindo audiometria tonal e vocal, VEMP e eletrococleografia extratimpânica. A sensibilidade e especificidade para detectar a presença ou ausência de doença foram calculadas e os respectivos intervalos de confiança de 95% obtidos. A confiabilidade dos testes de diagnóstico VEMP e eletrococleografia em ambas as orelhas foi avaliada pelo índice kappa.

**Resultados:**

Em ambos os testes e em ambas as orelhas, a capacidade para diagnosticar os casos saudáveis é alta, a especificidade variando de 84,6%–100%. Além disso, a capacidade dos testes para o diagnóstico da doença varia de baixa a moderada sensibilidade, com valores de 37,5%–63,6%. A concordância dos dois testes na orelha direita, medida pelo coeficiente de kappa foi igual a 0,54; com 95% IC (0,20–0,89), indicando uma concordância moderada. Para a orelha esquerda essa concordância foi igual a 0,07 com 95% IC (-0,33–0,46), indicando uma concordância fraca entre os testes. A sensibilidade do VEMP para a orelha direita foi de 63,6% e para a orelha esquerda, de 62,5%. A sensibilidade da ECochG para a orelha direita foi de 63,6% e 37,5% para a orelha esquerda.

**Conclusão:**

A especificidade de ambos os testes foi alta e a sensibilidade do VEMP foi maior que a da eletrococleografia.

## Introduction

Ménière's disease (MD) is an inner ear disorder characterized by episodic vertigo, tinnitus, ear fullness, and fluctuating hearing. The definitive diagnosis is made histologically, and can only be performed *post-mortem*, after analysis of the temporal bone. In 1995, the American Academy of Otolaryngology – Head and Neck Surgery (AAO-HNS)^1^ developed diagnostic criteria that are widely used. Recently, the Bárány Society developed a new guideline for the diagnosis of MD; after an evolutionary understanding of MD and vestibular migraine, the most common differential diagnosis, the need to update these criteria was highlighted.^2^

For a long time, it was believed that endolymphatic hydrops would be the histopathological substrate of the disease; this occurs more often in the cochlea and saccule, followed by the utricle and semicircular channels.^3^, ^4^ Recent studies have indicated that hydrops is a finding of the MD, together with the symptoms, since it alone does not explain all the clinical features, including the progression of hearing loss and the frequency of vertigo attacks. According to the criteria of the AAO-HNS, individuals with two or more spontaneous episodes of vertigo, lasting ≥20 min, with documented hearing loss in at least one occasion and tinnitus or ear fullness are clinically classified as having definite MD. The disease is called probable when a defined episode of vertigo in the presence of documented sensorineural hearing loss in at least one occasion, ear fullness, or tinnitus. MD is also classified as possible in the presence of Ménière-type episodic vertigo without documented hearing loss or when there is sensorineural hearing loss, fixed or floating, associated with imbalance, without a defined episode of dizziness.^1^

According to the criteria of the Bárány Society, MD is classified as definite or probable. In definite MD, the patient should have had two or more spontaneous episodes of vertigo, each lasting from 20 min to 12 h; documented mild to moderate sensorineural hearing loss; aural symptoms (hearing, tinnitus, and fullness) in the affected ear; and exclusion of other vestibular disorders that explain the symptoms. In probable MD, the patient should have had two or more episodes of vertigo or loss of balance, each lasting from 20 min to 24 h; floating aural symptoms (hearing, tinnitus, or fullness) in that ear; and exclusion of other vestibular disorders that explain the symptoms.^2^

The cause of hydrops is still unknown, and most theories are based on the change in production or resorption of endolymph. In 1982, Schuknecht^5^ postulated that hydrops causes rupture of Reissner's membrane, allowing the endolymphatic fluid, rich in potassium, to contact the perilymph and reach the surface of the ciliated cells and the vestibulocochlear nerve, causing hearing loss and vertigo attacks. Some believe that even the distension of the basilar membrane by the endolymphatic hydrops may already lead to degeneration of ciliated cells and therefore their malfunction, causing a decrease in the action potential (AP).^6^ Anatomical and vascular abnormalities are possibly related to its etiopathogenesis.

In 1989, Rauch et al. found histological evidence of endolymphatic hydrops in 13 out of 13 cases of patients with MD, but a review of medical records of six out of 19 temporal bones with endolymphatic hydrops revealed no signs or symptoms of MD. They observed that many inner ears have endolymphatic hydrops without clinical syndrome manifestation. Some suggest that endolymphatic hydrops may be an epiphenomenon of the pathophysiological mechanism of MD.^7^

The hypothesis of genetic predisposition is widely accepted, since a positive family history is present in many patients with MD. Research shows that the disease could result from mutations in the short arm of chromosome 6, where the histocompatibility antigen (HLA) is located; such mutations would be synergistic to the development of MD. Approximately 7% of patients with familial MD have an autosomal dominant model with 60% penetrance and a genetic pattern of anticipation, in which the next generation with the disease will present more intense symptoms with earlier onset.^6^, ^8^ Genetic studies in families suggest that the genetic mechanism associated with MD is complex and that there is more than one gene involved in most cases.^6^, ^8^, ^9^

As other inner ear disorders, MD has also been regarded as an autoimmune disease, as evidenced by a relationship with circulating immune complexes, suggesting deposition in the endolymphatic sac.^6^, ^9^ The autoimmune theory has also been strengthened by recent evidence of anti-endolymphatic sac auto-antibodies in the serum of patients with MD. Inhalant and food allergies have been associated with MD and, in many cases, allergic patients improved their symptoms after specific antiallergic therapy.^6^, ^9^, ^10^

Electrocochleography (EcochG) has been used for years in the diagnosis of endolymphatic hydrops in the cochlea. Its clinical application, however, is still controversial among otorhinolaryngologists because of its variable sensitivity, as in cases of hearing loss due to disease progression, patients may experience a reduction in the amplitude of AP due to loss of auditory nerve fibers.^11^, ^12^

Vestibular evoked myogenic potentials (VEMP) emerged as a method to assess vestibular function in 1994, when Colebatch and Halmagyi reported surface potential in the sternocleidomastoid (SCM) muscle in response to clicks through high-intensity air conduction (100 dB), accessing the sacculo-collic reflex.^13^, ^14^ These potentials assess the saccular and inferior vestibular nerve function, and are absent or decreased by 30–54% in patients with MD. The exam is easy to perform, does not cause discomfort to patient, and does not vary with hearing loss.^13^, ^14^, ^15^ VEMP can be obtained by air and bone conduction and galvanic stimulation, using tone burst or clicks as stimulus.^14^, ^15^, ^16^

The diagnosis of MD can be difficult, especially in cases where vestibular symptoms are present in isolation (vestibular MD).^17^ Until 1994, there was no unique method to assess saccular and inferior vestibular nerve function.^17^, ^18^ As the saccule is the second most prevalent affected site of endolymphatic hydrops, representing most forms of severe hydrops, VEMP appears as an auxiliary tool in the diagnosis of MD.^18^, ^19^

This study aimed to assess the sensitivity and specificity of VEMP and EcochG in the diagnosis of MD, as well as the degree of agreement between both tests.

## Methods

This was a clinical, prospective trial, which selected 12 patients (24 ears) diagnosed with definite MD according to the clinical criteria proposed by the AAO-HNS in 1995, of whom seven were females and five were male, aged between 33 and 63 years with a mean of 48.41 years; 12 healthy individuals, sex- and age-matched, were allocated to the control group (24 ears). The exclusion criteria were impossibility of cervical rotation and middle or external ear disease. The two researchers who conducted the assessments were unaware of the group to which the patient had been allocated.

The study was approved by the Research Ethics Committee under opinion No. 10668613.2.0000.0030. All participants signed an informed consent for inclusion in the study.

Clinical diagnosis by the AAO-HNS 1995 criteria was considered as the gold standard, and all patients underwent otorhinolaryngological and otoneurological examinations, including pure tone and speech audiometry, VEMP, and extratympanic EcochG.

Clinical history was taken and a physical examination was conducted; the differential diagnosis was excluded and the clinical diagnosis of MD according to the AAO-HNS 1995 criteria was confirmed.

### Tonal and speech audiometry/impedance

The examination was performed in all patients, with the following goals:

• Diagnose hearing loss wich is part of the definite MD diagnostic criteria by the AAO-HNS;
• Discard cases with middle ear diseases; and
• Discard cases with conductive loss that alters VEMP parameters.

### VEMP

**Figure 1:**
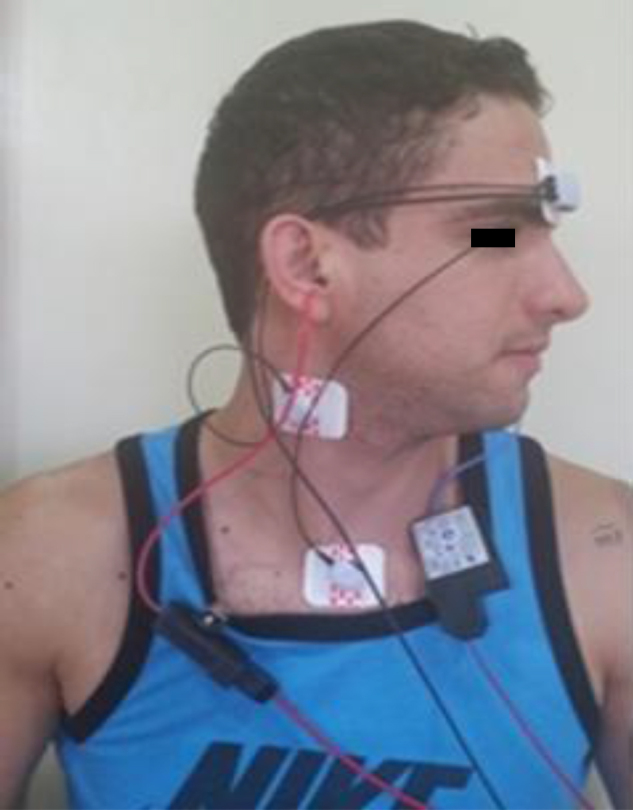
VEMP performed on the right.

The recording device was the Vivo *Sonic Integrity*, with programming for evoked potentials, using a specific protocol for performing the VEMP. Myogenic potentials are picked up by electrodes placed on the patient's SCM muscle (ipsilateral to the sound stimulus), the reference electrode (negative) is placed on top of the SCM; the active electrode (positive), on the sternum; and the ground, on the forehead. Patients were instructed to turn their head in the opposite direction to the sound stimulus, so that there was a contraction of the SCM muscle (Fig. 1).

#### Stimulus and response acquisition parameters

150 tone burst stimuli were used, at a frequency of 500 Hz, with the rate of 7.1 stimuli/s, stimuli intensity of 95 dB HL, high pass filters of 30 Hz and low-pass filters of 1000 Hz, presented through ER-A3 earphones. The recordings were made in a 30-ms window.

The following analysis criteria were considered: presence or absence of reproducible waves and interaural response asymmetry index for the amplitude.

The presence of reproducible waves and interaural response asymmetry index for the amplitude equal to or less than 34% characterized normal VEMP.

In turn, the absence of reproducible waves and/or interaural response asymmetry index for the amplitude greater than 34% characterized altered VEMP.

### EcochG

**Figure 2:**
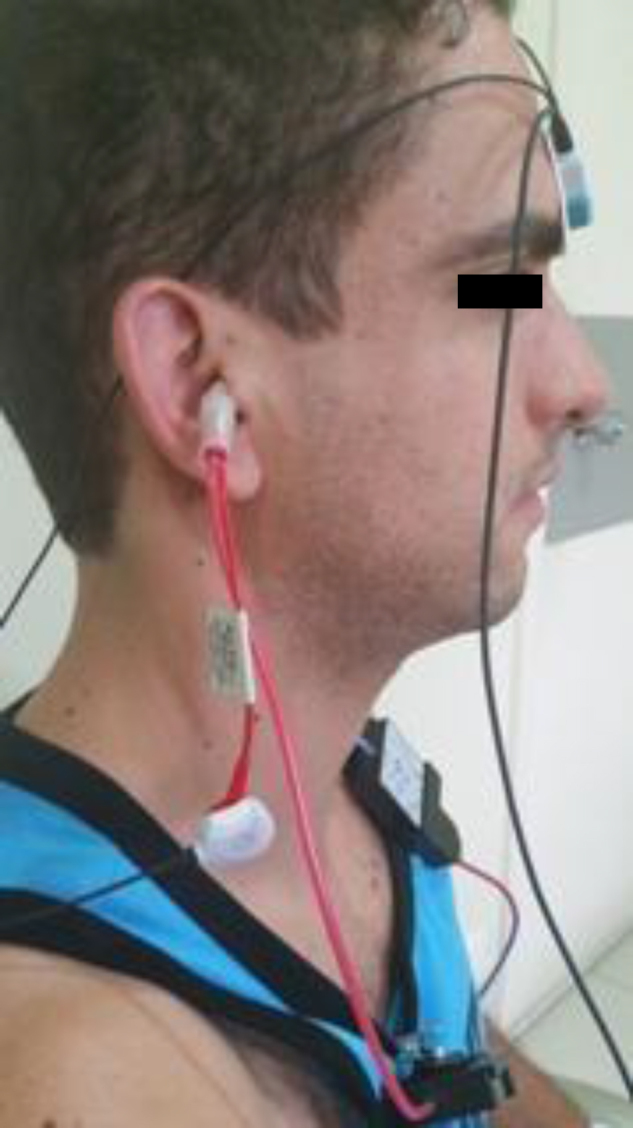
EcochG performed in the right ear.

The recording device was the Vivo *Sonic Integrity* and TIPtrode electrodes were inserted in the external auditory meatus. The ear canal was cleaned with abrasive paste. Negative and positive electrodes were connected to the TipTrode earphone; the negative electrode in the stimulated ear, the positive in the contralateral ear, and the ground electrode on the forehead (Fig. 2).

#### Stimulus and response acquisition parameters

Click stimuli were utilized (2–4 kHz), at a rate of 11.3 stimulus/s; intensity of 99 dB HL, high-pass filters of 30 Hz, and low-pass filters of 2400 Hz. The recordings were made with a 5-ms window.

An SP/PA ratio greater than 50% was considered altered.

### Statistical analysis

In order to validate both diagnostic tests (VEMP and EcochG), the clinical diagnosis was considered as the gold standard. Both ears were classified as with or without the disease, and as test instrument, the positive or negative result for the disease. Measures of sensitivity and specificity for the presence or absence of the disease were calculated, as well as their respective 95% confidence intervals. The reliability of diagnostic tests in both ears was assessed by kappa, using the scale proposed by Landis and Kock, which classifies the agreement as: (≤0, poor; 0.10–0.19, weak; 0.20–0.39, regular; 0.40–0.59, moderate; 0.60–0.79, substantial; 0.80–0.99, almost perfect; 1, perfect). The proportions of positive and negative results of the diagnostic tests were compared using McNemar's test. For data analysis, SAS 9.3 was used.

The significance level was set at *p* < 0.05.

## Results

At the VEMP, the mean p13 latency for the control group was 15.93 ms, with standard deviation (SD) of 0.85 ms. The mean n23 latency for the control group was 22.80 ms (SD = 1.16 ms). The mean asymmetry index for the control group was 16.22 (SD = 15.58). At the EcochG, the mean SP/AP ratio for the control group was 24.39% (SD = 11.61). All control group patients had pure tone and speech audiometry and impedance within normal limits.

In the study group, six cases of bilateral MD, five cases of unilateral MD in the right ear, and one case of MD in the left ear were identified, totaling 11 right ears and seven left ears. Regarding VEMP, 14 ears presented absence of waves and ten ears showed the presence of biphasic waveform; both ears showed the presence of waves in only three patients, thus enabling calculation of the asymmetry index. The mean asymmetry index for these patients was 11.22. The mean SP/AP for the case group was 47.23.

Table 1, Table 2 show the individual results of the study group on the right and left ears, respectively. Table 3, Table 4 show the individual results of the control group on the right and left ears.

**Table 1:** Individual results of the right ear in the case group.

Case group right ear					Impedance
Patient	Clinical DT	VEMP	EcochG	Audiometry	
1	Yes	Normal	Normal	Moderate SNHL, ascending	A curve
2	Yes	Altered	Altered	Mild SNHL, flat	A curve
3	No	Altered	Normal	Normal	A curve
4	Yes	Altered	Altered	Moderate SNHL, flat	A curve
5	Yes	Altered	Altered	Mild conductive hearing loss	A curve
6	Yes	Normal	Altered	Moderate SNHL, descending	A curve
7	Yes	Normal	Altered	Mild SNHL, flat	A curve
8	Yes	Normal	Normal	Mild SNHL, flat	A curve
9	Yes	Altered	Normal	Moderate SNHL, descending	A curve
10	Yes	Altered	Altered	Moderate SNHL, descending	A curve
11	Yes	Altered	Altered	Mild SNHL, inverted U	A curve
12	Yes	Altered	Normal	Mild SNHL, descending	A curve

**Table 2:** Individual results of the left ear in the case group.

Case group left ear					Impedance
Patient	Clinical DT	VEMP	EcochG	Audiometry	
1	Yes	Normal	Altered	Mild SNHL, flat	A curve
2	No	Normal	Normal	Normal	A curve
3	Yes	Altered	Normal	Mild SNHL, flat	A curve
4	Yes	Normal	Altered	Moderate SNHL, flat	A curve
5	No	Altered	Normal	Normal	A curve
6	No	Normal	Normal	Normal	A curve
7	No	Normal	Normal	Normal	A curve
8	Yes	Altered	Normal	Moderate SNHL, ascending	A curve
9	Yes	Altered	Normal	Moderate SNHL, descending	A curve
10	Yes	Altered	Normal	Moderate SNHL, descending	A curve
11	Yes	Altered	Altered	Moderate SNHL, flat	A curve
12	Yes	Normal	Normal	Mild SNHL, descending	A curve

**Table 3:** Individual results of the right ear in the control group.

Control group right ear					Impedance
Patient	Clinical DT	VEMP	EcochG	Audiometry	
13	No	Normal	Normal	Normal	A curve
14	No	Normal	Normal	Normal	A curve
15	No	Normal	Normal	Normal	A curve
16	No	Normal	Normal	Normal	A curve
17	No	Normal	Normal	Normal	A curve
18	No	Normal	Normal	Normal	A curve
19	No	Normal	Normal	Normal	A curve
20	No	Normal	Normal	Normal	A curve
21	No	Normal	Normal	Normal	A curve
22	No	Altered	Altered	Normal	A curve
23	No	Normal	Normal	Normal	A curve
24	No	Normal	Normal	Normal	A curve

**Table 4:** Individual results of the left ear in the control group.

Control group left ear					Impedance
Patient	Clinical DT	VEMP	EcochG	Audiometry	
13	No	Normal	Normal	Normal	A curve
14	No	Normal	Normal	Normal	A curve
15	No	Normal	Normal	Normal	A curve
16	No	Normal	Normal	Normal	A curve
17	No	Normal	Normal	Normal	A curve
18	No	Normal	Normal	Normal	A curve
19	No	Normal	Normal	Normal	A curve
20	No	Normal	Normal	Normal	A curve
21	No	Normal	Normal	Normal	A curve
22	No	Normal	Normal	Normal	A curve
23	No	Normal	Normal	Normal	A curve
24	No	Normal	Normal	Normal	A curve

**Figure 3:**
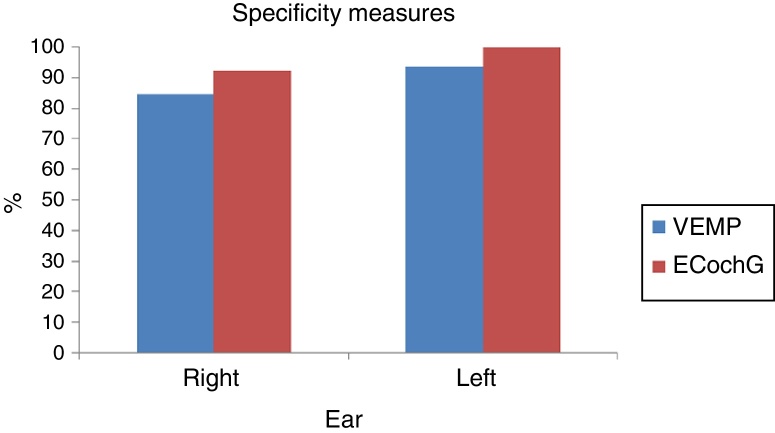
VEMP and ECochG specificity in the right and left ears.

**Figure 4:**
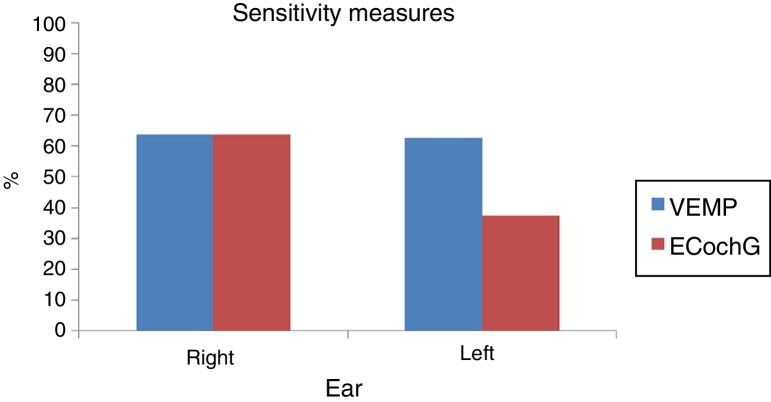
VEMP and ECochG sensitivity in the right and left ears.

In both tests and both ears, the ability to diagnose the healthy cases was high: the specificity ranged from 84.6% to 100% (Fig. 3). Moreover, the ability of the test to diagnose the disease varied from low to moderate, which a sensitivity of 37.5–63.6% (Fig. 4). Table 5 shows the sensitivity and specificity of both tests in both ears.

**Table 5:** Sensitivity and specificity values, by type of test in both ears.

Ear	Diagnostic test	Clinical examination			
		Diseased		Healthy	
		*n*	% (95% CI)	*n*	% (95% CI)
**Right**	**VEMP**				
	Positive	7	63.6 (30.8–89.1)	2	–
	Negative	4	–	11	84.6 (54.6–98.1)
	**EcochG**				
	Positive	7	63.6 (30.8–89.1)	1	–
	Negative	4	–	12	92.3 (64.0–99.8)

**Left**	**VEMP**				
	Positive	5	62.5 (24.5–91.5)	1	–
	Negative	3	–	15	93.7 (69.8–99.8)
	**EcochG**				
	Positive	3	37.5 (8.5–75.5)	0	–
	Negative	5	–	16	100.0

The agreement of both exams in the right ear, measured by the kappa coefficient, was equal to 0.54 (95% CI: 0.20–0.89), indicating a moderate agreement. In the left ear, that agreement was equal to 0.07 (95% CI: −0.33 to 0.46), indicating a weak correlation between the tests (Table 6).

**Table 6:** Agreement between diagnostic tests.

Ear	VEMP	EcochG			Kappa (95% CI)
		Positive	Negative	Total	
**Right**					0.54 (0.20 to 0.89)
	Positive	6	3	9 (37.5)	
	Negative	2	13	15 (62.5)	
	Total	8 (33.3)	16 (66.7)	24 (100.0)	

**Left**					0.07 (−0.33 to 0.46)
	Positive	1	5	6 (25.0)	
	Negative	2	16	18 (75.0)	
	Total	3 (12.5)	21 (87.5)	24 (100.0)	

**Figure 5:**
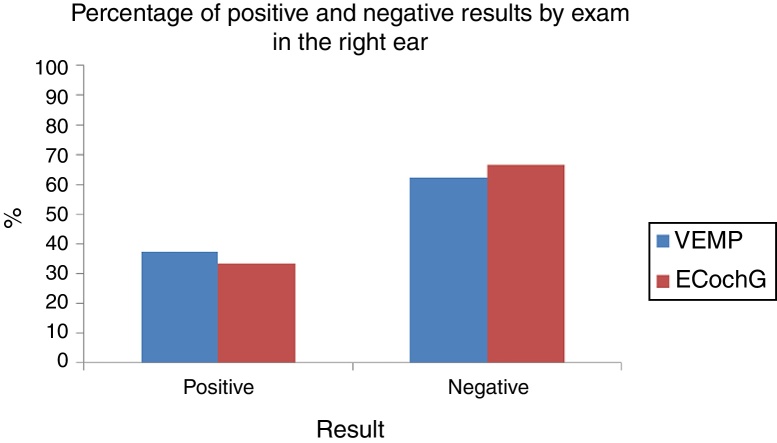
Percentage of positive and negative results by exam in the right ear.

**Figure 6:**
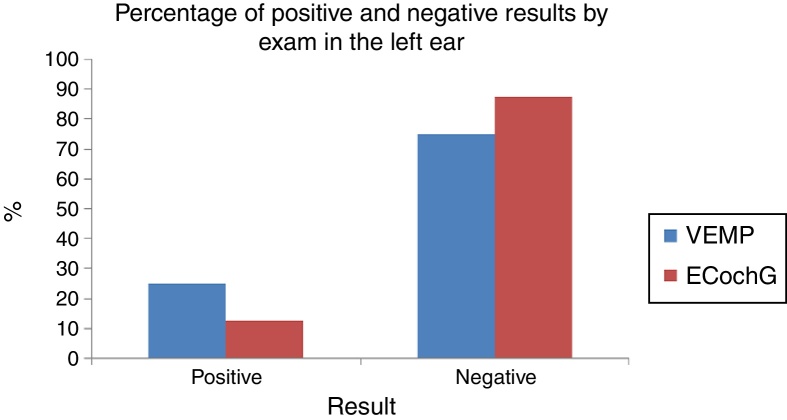
Percentage of positive and negative results by exam in the left ear.

For the right ear, the proportion of positive and negative test VEMP results (37.5–62.5%) did not differ significantly from the proportion of positive and negative results in EcochG (33.3–66.7%; *p* = 0.6547; Fig. 5). For the left ear, the proportion of positive and negative test VEMP results (25.0–75.0%) did not differ significantly in the proportion of positive and negative results in EcochG (12.5–87.5%; *p* = 0.2568; Fig. 6).

## Discussion

12 patients with definite MD and controls were evaluated by audiometry and impedance, VEMP, and EcochG.

In the present study, VEMP was performed in a sitting position, which according to previous studies is the position that provides better activation of the SCM.^20^, ^21^, ^22^ Tone burst stimuli were used, since stimuli with frequencies near 500 Hz have higher response amplitudes.^23^ The large variation in response amplitude caused by different degrees of muscle contracture obtained for each individual justifies the analysis of the VEMP responses through the interaural asymmetry index. Based on a review of the literature, values above 34% were considered as altered.^22^, ^24^ However, a biphasic p13-n23 wave was observed in ten ears (41.66%), whereas only three patients showed the presence of waves in both ears, allowing for the calculation of the asymmetry index. In 1999, Seo et al.^18^ observed biphasic waves in 72% of cases of MD and Waele,^17^ in 45.7% of cases.

14 ears (58.33%) showed no waves at VEMP. The lack of response, as well as asymmetry index greater than 34%, suggest endolymphatic hydrops.^17^, ^18^, ^24^ Jariengprasert et al.^25^ observed that the absence of waves and asymmetry index alterations were more significant than P1 and N1 latencies or amplitude measures in the identification of saccular dysfunction in MD.

Depending on the severity of hydrops, some patients may present irreversible degeneration of the sensory epithelium of the saccular macula, with absence of waves. In 1999, Waele^17^ also attributed the lack of VEMP response in patients with MD to insufficient contraction during the examination, unrecognized vestibular pathology, or saccule hyposensitivity due to the aging of the saccular macula in the elderly. This ineffective contraction can be avoided by using electromyography to monitor the degree of muscle contraction, which was not possible in the present study, since the equipment used did not have this feature.

The EcochG used in this study was extratympanic, which, according to the literature review, is an effective and non-invasive method of measuring cochlear hydrops.^26^ The presence of SP and AP was observed in all patients.

The responses showed a low to moderate sensitivity of VEMP and EcochG in the diagnosis of MD in relation to clinical diagnosis. The sensitivity of the VEMP for the right ear was 63.6% and for the left ear, 62.5%. It was slightly higher than that found in the literature, which ranged from 40% to 54%.^16^, ^17^, ^27^ The sensitivity of EcochG for the right ear was 63.6% and for the left ear, 37.5%; the literature values range from 57% to 71%.^26^

The fluctuating course of the disease complicates the interpretation of electrophysiological tests. The main question regarding the use of diagnostic tests in MD pertains to their sensitivity. Egami et al.^14^ observed that, although the VEMP sensitivity was not high, it was comparable to the caloric test, providing additional information to identify vestibular abnormalities in MD.

The high specificity of both tests was consistent with findings in the literature, suggesting their high accuracy in ruling out the presence of disease, assisting especially in cases where there is differential diagnosis.^14^, ^26^

Regarding the VEMP, two asymptomatic ears had changed VEMP. This finding is described in the literature as a result of occult saccular hydrops or of binaural interactions in the otolith-cervical reflex arc of VEMP. Similar findings were retrieved in the literature.^15^, ^16^, ^17^, ^18^ In 2006, Lin et al. found that 27% of patients with unilateral MD showed high thresholds in the asymptomatic ear, demonstrating that VEMP can be altered even before the appearance of the classic symptoms of the disease, and that saccular hydrops may precede the symptoms of bilateral MD.^19^, ^28^, ^29^ In 2000, Conlon and Gibson^30^ also reported abnormal clinical findings in asymptomatic ears. There are also individual differences in the degree of muscle tone and contraction,^28^, ^31^ which the authors of the present study tried to avoid by sex- and age-matching the control group and by using the interaural asymmetry index as a parameter.

The ability to predict the presence of abnormalities in an asymptomatic ear is one of the great features of VEMP. Knowing whether the patient has the disease in the contralateral ear, for example, aids in making a decision about ablative procedures in the diseased ear.^28^

Seven ears with MD had normal VEMP. These ears may have saccular macula free from hydrops, presenting cochlear hydrops. In 1987, Okuno and Sando^3^ examined 26 temporal bones of patients with MD and demonstrated that endolymphatic hydrops was observed most frequently in the cochlea, followed by the saccule, utricle, and semicircular canals. The literature also reports that many of the cases of severe hydrops refractory to medical treatment may be located in the saccule.^16^

Regarding EcochG, no asymptomatic ear had altered values, and nine ears with MD had normal values. Of these ears, five had moderate sensorineural hearing loss and four had mild sensorineural hearing loss. Audiometric thresholds around 50 dB undermine the analysis of hydrops by EcochG,^32^ and are therefore a hypothesis for the normal EcochG observed in five patients with moderate hearing loss. The literature also reports that patients with hydrops in otolith organs may have disease-free cochlea, which is another hypothesis that could apply to patients with mild hearing loss. A major advantage of VEMP is that the saccular macula is sensitive to sound even after total destruction of the cochlea, and thus it can be performed in individuals with good or bad hearing acuity.^14^, ^17^, ^19^

The sample size was one limitation of the present study. Definite MD, with document hearing loss, it is not highly prevalent, hindering the selection of patients for the study. For the same reason, it was not possible to compare patients with the same disease duration. Ears with longer duration of symptoms show higher abnormalities in the SP/AP ratio, and once the SP rises, it persists for long periods^26^, ^33^; that is, even in the period between attacks the EcochG can demonstrate cochlear hydrops.

Cervical VEMP by air conduction may be increased in the early stages of MD, perhaps due to the pressure of saccular hydrops against the stapes footplate, increasing saccular sensitivity to intense sound.^16^ Its measurement can be variable, with a tendency to disappear with the progression of the disease, as well as during the 24 h post-crisis, and may reappear after 48 h or with the use of drugs to reduce endolymphatic hydrops.^2^, ^11^, ^13^, ^34^

The agreement measured by the kappa coefficient evaluates the similar results between both exams. The low correlation between the diagnostic tests was expected, as they evaluate different structures. Furthermore, they have different sensitivities according to the stage of the disease; Vemp is more altered in the symptomatic period, unlike Ecochg, that even in the period between attacks can demonstrate cochlear hydrops. The agreement for the left ear was low, and lower than that for the right ear, probably because the size of the left ear sample was smaller than that of the right ear. McNemar's test assessed the proportion of positive and negative results of VEMP and EcochG, which were not statistically different from each other, both in the right and left ears.

In the control group, the subjects were sex- and age-matched. However, further studies could pair the control group for hearing loss similar to those of patients in the case group instead of selecting controls with normal hearing, since the hearing thresholds are determining in the sensitivity of EcochG.

The possibility of assessing saccular hydrops through a noninvasive, easy to perform method, such as the VEMP, aids the otorhinolaryngologist in disease management. In 2001, Murosfushi reported that the prolongation of latency in VEMP suggests retrolabyrinthine injury, facilitating the exclusion of differential diagnoses.^25^

Several factors corroborate the aid of additional tests, such as VEMP and EcochG, in the diagnosis of MD. Possible MD cases in which there is no documented hearing loss, the heterogeneity of the disease, and the involvement of various labyrinthine structures often complicate the diagnosis. VEMP and EcochG are complementary tests, since they assess different labyrinthine structures, and should be incorporated into routine otoneurological examinations, aiding in the identification of hydrops location and the possibility of asymptomatic ear involvement.

The assessment of cochlear function with audiometry and EcochG, of the saccule through cervical VEMP, of the utricle through ocular VEMP, of the lateral semicircular canal through caloric tests, and of all the semicircular canals through vHIT demonstrates the advancement of research in the vestibular diagnosis. New paths are open to the discovery of the pathophysiological mechanism of a disease that was first described over a century ago and still has no defined treatment protocol.

## Conclusion

The specificity of both tests was high, and the sensitivity of VEMP was higher than that of EcochG.

## Conflicts of interest

The authors declare no conflicts of interest.
